# What Medicine Is Accomplishing Today1Anniversary address of the president of the Medical Society of the State of New York. Delivered in the Senate Chamber at Albany, January 31, 1905, during the ninety-ninth annual meeting.

**Published:** 1905-03

**Authors:** Hamilton Dox Wey

**Affiliations:** Elmira, N. Y.


### BUFFALO MEDICAL JOURNAL.

VOL. XLIV.—LX. MARCH, 1905. No. 8

### ORIGINAL COMMUNICATIONS.

### What Medicine is Accomplishing Today.[1]

By HAMILTON DOX WEY, M. D., Elmira, N. Y.

THE practice of the medical profession, when brought to the attention, other than through the common round and daily task, as by the medium of narrative and pen picture, quickens the pulse and increases its tension as the story is unfolded of the struggle with disease and amelioration of human suffering. I take exception to the frequently expressed and ill-considered statement that contact with misery and familiarity with the morbid processes we denominate disease blunt the sensibilities of the physician, rendering him insensible to distress of mind and body and unmindful of effects immediate and remote upon the individual, the family, and even the community itself.

From disease successfully combatted, life spared and prolonged through surgical intervention, there accrue a satisfaction and pleasure transcending personal considerations. Much as we may affect brusqueness of manner and force into the background expression of sentiment and feeling, there is woven into the woof and warp of our composition, the feelings, emotions and sentiments tending to and constituting the ethical condition termed altruism.

In consonance with this, professional life, reflected in the mirror of romance, strengthens the impulses and perceptions, endearing to us,—if such a thing be possible,—our profession, and, in the presentation afforded of the combined view of patient and physician, quickens our thoughts in connection with subjects it is fitting we should scan at times, revealing the physician as

1. Anniversary address of the president of the Medical Society of the State of New York. Delivered in the Senate Chamber at Albany, January 31, 1905, during the ninety-ninth annual meeting.

we would have him appear, and as we ourselves would impress ourselves upon those with whom we come in contact.

I regard it as not sentimentalism, but the evidence of deep feeling and tender touch with the seriousness of living, that the story of "Rab and his Friends" and the tale of "A Doctor of the Old School" should occasion a dimming of vision in their perusal. It is a distinction of which the land of the heather may well be proud that two such pen pictures should have originated within her border. Reference is due to Balzac's "Country Doctor," and the list might be multiplied of works descriptive of the practitioner of medicine in remote regions and under trying circumstances typifying progressive and advanced medicine.

But the tragedy and pathos of medicine are not confined to narrative and pen tracing. Along the skirmish line of militant medicine, in advance of the rank and file of the profession, occur incident and happening surpassing in tragedy and emotion anything presented in mimic life. No general upon the battlefield or soldier pressing forward to rescue the falling colors of his regiment, exhibited greater bravery and more complete indifference to personal safety than did Dr. Ephraim McDowell in the performance of his first ovariotomy upon that memorable day of 1809. The knowledge an excited crowd of men were waiting without to do him violence, even to the taking of his life in the event of Mrs. Crawford dying upon the table, did not deter him from the performance of his duty, as it appeared to him, nor rob his hand of its skill and cunning.

The illustrious names in medicine and surgery that have come down to us through succeeding years are those of men in the skirmish line or in the front ranks of advancing medicine, and far removed from rank and file. They appear with peculiar and particular distinctness in the history of medicine, and with a distinction attaching to them different in kind from that enjoyed by those famous in the profession today.

There are several reasons for this, among which, I opine, are the times in which they lived, their personality emphasised by separation from one another, and the particular and distinct benefits they conferred upon medicine. In their day there was not the communication, contact and parallel lines of research that characterise the present, but to a greater or less degree each advance made and truth declared was sporadic in character. Today research, revelation and advancement is epidemic,—it is windborne,—so that he who reads must run.

The application of Ambrose Pare in 1552 of the ligature to vessel ends in amputation; the work of Jenner in connection with vaccination and preventive medicine; association of the Chamberlains with the obstetrical forceps, (albeit their fair fame was clouded by their secrecy and unethical conduct); devising the stethoscope by Laennec; advancement made by Graves in the treatment of febrile diseases and the distinction, "He fed fevers," attaching to him; the efforts of Pinel for a humane and merciful treatment of the insane are cited as instances of outposts of militant medicine. The citations are few, offered without intent of invidious distinction or discrimination against the fair fame of others equally illustrious. Your knowledge of medical history will readily supply omissions.

Coming down to more recent date, we find ourselves under the immediate influence of the achievements of Morton in the production of ether anesthesia; Sir James Y. Simpson's discovery of the anesthetic properties of chloroform and its applicability to the alleviation of pain during childbirth; Holmes's declaration of the contagiousness of puerperal fever; the efforts, research and investigation of Virchow and his pupils to render medicine into an exact science through the application of applied methods; the contributions to and labors of Sims and Emmet in the field of gynecology; materialness of Lister upon surgery and surgical technic of today arising from his appreciation of the import of wound infection, and the researches of Pasteur and Koch to elucidate and demonstrate the germ nature of infectious disease.

Without being bound to or obstructed by precedent, the trend of professional life is onward. The theory and practice of today have superceded that of yesterday and will, in turn, be displaced by that of tomorrow. Blind adherence to the dogma and methods laid down by college days is no longer in order. The practitioner, to retain his prestige and preserve professional influence in the community in which his lines are cast, must join and be one of the procession moving onward. The camp follower is discredited and the man who stands still in his professional tracks unswervingly committed to methods of the past and a generation ago, impairs his usefulness and renders himself a reminder of other days.

Individual limitation relegates many physicians, and the same is true of other walks of life, to the plane of mediocrity. Many, susceptible of professional betterment and capable of occupying a higher position than the one they fill, are prevented by family and other considerations, readily suggesting themselves, from participation in the educational advantages and opportunities now offered the practitioner.

But despite the low terms of personal equation, evidenced by inflexible routine practice, acquaintance with medical literature, chiefly through the medium of proprietary journals, and the absence of spirit that permits of the application of the appellation "Doc"; also the necessitous circumstances incident to practise in a sparsely settled and rugged country, barely yielding a present living and without affording future provision for dependent ones; the influence of progressive medicine and the reflex of advanced thought are felt and manifest with resultant conferring upon each community and every locality an increased standard of ordinary medical skill.

This applies equally to remote sections, where surgery is practised with a fearlessness and in kind that a few generations since would have been associated with most illustrious names. In glen and upland, far removed from the conventionalities and polish of city life, weather-beaten, prematurely aged and furrowed men, with touch as delicate and tender as that of fond mother cooing over her laughing babe, are performing hysterectomies, removing diseased ovaries and operating in cases of septic appendicitis. And by no means do all their patients die. Patients sometimes die in cities! But to such men at times, in moments of retrospection, after a difficult case, carrying with it perplexity, anxiety and uncertainty as to the outcome, the sentiment uttered by the "Doctor of the Old School", "A' did what a' cud to keep up wi' the new medicine, but a' had little time for readin' and nane for travelin,' " is felt if not expressed.

It is a privilege to have practised medicine during the decade last passed and the years immediately preceding. Within this period the "germ theory of disease", dreamed of and speculated concerning by the physician of a generation and longer ago, has become an established fact. I say established fact, but with the reservation that while the *status presens* of our knowledge has not yet reached the stage of recognition, isolation and culture of particular protozoa and bacteria associated with each morbid entity, yet, nevertheless, the advance made and benefits accruing to the practice of medicine at the present time from laboratory methods and research hold forth the reasonable expectation the near future will reveal the true character of the materies morbi of most, if not all disease, the manner of propagation and transmission and indicate scientific and exact procedure of treatment in place of an expectant and empirical plan.

From the demonstration by Koch in 1882 of the bacillus tuberculosis to the present time is comprised a period of revolutionary medicine. Etiology of disease, pathology, treatment, preventive medicine, the domain of medicine and surgery, the welfare of mankind have been incalculably benefited by the researches of those, our associates, who, while not generally engaged in the actual practice of medicine, have conferred alike upon patient and physician benefits and increased capacity for well doing transcending calculation and description.

I refer to the laboratory workers,—biologists, bacteriologists, hematologists. In response to their tireless, never ceasing activity, new terms and expressions have multiplied and an almost new medical nomenclature. Successive editions of standard works relegate their predecessors as curiosities to upper shelves of the library. Almost all that remains of use or value to us in the writings of the pastmasters of medicine is their classic language and the accuracy of description of the objective phenomena of disease.

It has been tersely stated: "Science does not demand practical results, but investigates for the purpose of establishing facts and explaining phenomena." Preconceived ideas do not always develop actualities, and established facts not infrequently have their origin in investigation seeking other facts than those obtained.

The discovery of the Klebs-Loeffler bacillus antedated Behring's serum eight years and its employment in the treatment of diphtheria. Here a little and there a little, established fact after fact, singly of comparatively little value, or only suggestively so, in conjunction become of greatest importance as exerting an influence upon the material prosperity and economic conditions of lands and nations in the elucidation of the nature of disease, agent of communication, prophylaxis and treatment.

Nearly twenty years intervened between the discovery by Laveran of the parasite of malaria and the conclusion of Celli: "That up to now the certain vehicle, and at the same time the certain source of the malarial infection, are special mosquitoes and the air is the vehicle of malaria, inasmuch as it is the vehicle of malarial mosquitoes."

I repeat, it is a privilege to practise medicine in these latter days, to have participated in whatever degree in the medicine of the last two decades. It is not permitted all to be original workers, to be identified with signal advance or achievement in medicine or surgery, but to each is permitted to maintain the dignity and reputation of the profession by adaptation to the methods of today,—use of diphtheria culture for the purpose of establishing diagnosis; the Widal reaction in doubtful or suspected cases of typhoid fever; blood examination in appropriate cases. The list might be further multiplied. It is unnecessary, however, so to do. The profession, generally, in close file, is marching onward, but there are following stragglers. It is incomprehensible there should be at this day those who shrink from the administration or question the efficiency of diphtheria antitoxin, a remedy that has reduced the mortality of this once more than now dreaded disease to approximately 2 per cent. when given during the first twenty-four hours.

The flitting years comprised in the professional activities of many of us have witnessed the revolutionising of surgery through antispetic measures and asepsis, identification of the bacillus of tuberculosis, typhoid fever, diphtheria, tetanus, anthrax, rabies and other conditions. Serum therapy, which, while not meeting wild expectation in every instance, in results and applicability has established itself as an indicated means of treatment in proper cases. Disappointment has been expressed, as in typhoid fever immunisation and the serum treatment of this disease, but failure in certain lines emphasises success in others, and stimulates expectation as to what the future has in store.

Whatever the future contains of promise, there is compensation in present living and relation to the near past, by reason of the Röntgen ray in diagnosis and treatment; the Finsen and other lights in therapeutic relation; intubation, through which the name of O'Dwyer is held in blessed memory, superceding that disappointing operation of last resort,—tracheotomy; the rôle of insects in the dissemination of disease, as flies of typhoid fever, emphasised during the Hispano-American war; the relation of special mosquitoes to malaria and yellow fever. It is impossible to estimate the benefits conferred and yet to accrue to countries and commerce through the study of this phase of insect life, in the prosecution of which Reed, Lazear and Graham laid down their lives in the fulness of their strength and intellect. "Greater love hath no man than this, that a man lay down his life for his friends."

For the first time in history, war is being waged with regard to preventive medicine. Japan is demonstrating to the world disablement and death from disease can be made to fall far below that arising from contact and the implements of war,—a reversal of past experience. So broad the field of research and many and varied the benefits accruing to medicine and surgery, voice fails and pen halts in attempted narrative and particularising. Each in his own sphere, general practitioner and specialist, has his energies and attention taxed in his efforts to keep abreast with that most closely related to the phase of medicine with which he is identified.

It is manifestly impossible for any one mind to master all problems of medicine and surgery. But, nevertheless, and especially in the case of the busy practitioner, there is unconscious acquiring of knowledge of what is new and progressive in medicine. Throughout the state, in villages and smaller cities, hospitals have multiplied schools for postgraduate instruction, a feature to the end the practitioner is enabled to familiarise himself with the new medicine, improve his operative technic and quicken diagnostic skill.

The hospitals referred to, in addition to primal purpose, are educational factors in their respective localities. They confer benefits upon members of their staff, contributing to the latter's experience and skill and enlarging the field of their activities. As many benefits accrue to the staff as the staff renders the hospital, and in the conferring of these benefits and privileges, the standard of medicine in a given locality is enhanced. Every community is benefited in the broadest meaning of the term by a hospital, and every physician connected therewith is a better, broader and more useful man by reason of such connection. The relation of smaller hospitals in rural and semiurban districts to schools of postgraduate instruction is a reciprocal one, each benefiting the other, both an earnest of scientific, precise medicine, and the influence of both, year by year more manifest and widespread. Both alike stand for higher education and rational medicine.

There is much to learn from European custom, which permits the savant, grown gray and proficient in his life's work, to continue uninterruptedly the study of the various problems of health and disease under government auspices, secure in his position, and without embarrassment of study on account of concern for the matters of everyday life.

Private largess is encouraging more and more research and elucidation of the medical questions of the day, but, unless in the form of endowment sufficient for the prosecution of specific inquiry, may prove capricious or pass with the death of an individual. Is the state discharging its obligation in the premises? The reply is not unreservedly in the affirmative. The bounty of the state is extended with liberal hand on account of pleasure and palate, in the betterment of the product of the farm and field, safeguarding from causes inimical to them, and in afforded opportunity for sport in forest and stream. But does it afford capacity for adequate enjoyment of these in conserving of the public health?

The sum, approximately, of $85,000 is available for all purposes in connection with the work of the state department of health: protection of potable waters, investigation of alleged nuisances, maintenance of cancer, antitoxin, pathological and bacteriological laboratories, the bureau of chemistry and registration of vital statistics; 12 per cent. of the appropriation of 1904 for agricultural and correlated objects and 27 per cent. of appropriation on account of forest, fish and game commission. In other words, the state expends annually 1.17 cents per capita for purposes pertaining directly to public health. Had it been necessary, an equal amount expended in the preparation and distribution of antitoxin to public and charitable institutions and for treatment and immunisation of the indigent and needy, those whose circumstances do not permit of the purchase of the remedy, would have been a public economic measure.

Aside from the work in hand and done, there is another phase of an active state department of health dealing with the immediate questions of the day and its influence upon the public at large, worthy of mention. Each diphtheria culture made, Widal test and examination of suspected water for evidences of sewage pollution, are educational factors and create in the various localities whence originating a sentiment favorable to laboratory methods, and greater precision in diagnosis. But the distance from various parts of the state and the time required for the receipt of reports of bacteriological and other examinations is often too great to allow the physician awaiting returns from a central bureau to verify diagnosis and indicate a plan of treatment. The factor of time is of importance and treatment in the interim must be instituted.

It would appear the time has arrived for each county to afford laboratory facilities. There should be a county bacteriologist to make blood counts and examinations, diphtheria cultures, Widal tests and examinations for pathologic bacteria, as the bacillus tuberculosis and the micrococcus of Niesser, and perform routine laboratory work, as related to general every-day practice of medicine. There are county officers connected with and of less importance to public weal than would be one of the character just suggested. Cities and many of the larger villages provide for themselves in this respect, and thus, in a measure, meet the demands of immediately contiguous territory.

County laboratories should not be regarded as an accommodation or concession to the profession, but occupying a higher plane, a benefit to the community in its entirety, without relation to class. Blood count and examination, interrogation of excretory and secretory products, cultures and laboratory work in general, pertain specially to no school or dogma of medicine, but are the conferred benefits of science and progression, applicable alike to patient and physician and conducing to the common welfare.

The establishment of county laboratories would relieve a central department of much routine work, emphasise the value of the latter as an advisory center, and confer greater latitude and opportunity for original investigation and research than obtains under existing conditions.

It may be asked, with the establishment of county laboratories, whence are to come those qualified and equipped to perform the duties in connection therewith? The spirit of the times, the incentive to onward march, has reached regions remote from medical and teaching centers. The law of demand and supply everywhere obtains. Once the demand created, I believe immediately or in brief time would be found those qualified to undertake work of the kind referred to. There are those skilled in microscopy and competent to engage in labors of the character under description, and others proficient in the use of the microscope, but not altogether grounded in laboratory methods. The former are available, and the latter, with prospect of an appointment, would, in comparatively brief time, qualify through special study and instruction to assume duties of the kind referred to, or as soon as the establishment of such an office could be provided.

The problem appears one easy of solution: the difficulty is in the initiation. Professional opinion and influence can alone create public sentiment favorable to progressive medicine. A double obligation rests upon the profession: the attaining of high standards and the education of the public thereto.

359 Main Street.

